# Pure Air

**Published:** 1884-04

**Authors:** 


### PURE AIR.

NO TIME could be better than the present for beginning the practice of house ventilation by the window, which is still, in the majority of houses, the readiest and the safest means of obtaining a regular and constant supply of fresh air. This practice, begun in warm weather, may be carried on with proper care, through autumn and winter. The constantly-accumulating impurities derived from the breath, from perspiration, from excreat of all other kinds derived from sleeping rooms, from the use of gas or lamp light—and too often, even now, from suction of sewage gas from waste pipes, by the heat of house fires, etc., render it as necessary for comfort that these should have free egress and that they should be substituted by pure outer air.

Fresh air from without may very easily be had without draught and without risk of cold even to delicate persons, if a few simple rules be observed. The cold air of winter of course enters with greater force and in greater proportional volume than the more equable summer air, into a warm room. The aperture of ingress must be correspondingly diminished. Air from a window is preferable to that from an opened inner door, no matter how roomy the house, from its more reliable purity.

